# Supplementary material for: A cofactor-promiscuous HMGR from the Lyme disease pathogen illuminates diversity in bacterial isoprenoid biosynthesis
Source: bioRxiv. 2026 Jul 7:2026.07.06.735745. Preprint. [Version 1] doi: 10.64898/2026.07.06.735745 (PMC13370337; doi:10.64898/2026.07.06.735745)
Supplement: Supplement 1 [file NIHPP2026.07.06.735745v1-supplement-1.pdf]

bioRxiv preprint doi: https://doi.org/10.64898/2026.07.06.735745; this version posted July 7, 2026. The copyright holder for this preprint (which was not certified by peer review) is the author/funder, who has granted bioRxiv a license to display the preprint in perpetuity. It is made available under a CC-BY 4.0 International license.

Movie legends

Movie S1: Video of *B. burgdorferi* cells (control) growing and dividing on a BSK-II agarose pad. Time-lapse phase-contrast microscopy of representative uninduced CJW_Bb662 cells.

Movie S2: Video of *B. burgdorferi* cells undergoing HMGR depletion on a BSK-II agarose pad. Time-lapse phase-contrast microscopy of representative CJW_Bb662 cells experiencing HMGR depletion on a BSK-II agarose pad containing the CRISPRi inducer IPTG.

bioRxiv preprint doi: https://doi.org/10.64898/2026.07.06.735745; this version posted July 7, 2026. The copyright holder for this preprint (which was not certified by peer review) is the author/funder, who has granted bioRxiv a license to display the preprint in perpetuity. It is made available under a CC-BY 4.0 International license.

Supporting Information for

A cofactor-promiscuous HMGR from the Lyme disease pathogen illuminates diversity in bacterial isoprenoid biosynthesis

Isaac A. Paddy,[1,2] Joshua McCausland,[2,3,4] Madelyn Frazier,[2,3,4] Poulami Chatterjee,[2,5] Mekedlawit Setegne,[2,5] Oliv Eidam,[6, #] Christine Jacobs-Wagner,[2,3,4,7] & Laura M. K. Dassama[2,5,7*]

[1]Department of Chemical and Systems Biology, Stanford School of Medicine

[2]Sarafan ChEM-H Institute, Stanford University

[3]Department of Biology, Stanford University

[4]Howard Hughes Medical Institute, Stanford University

[5]Department of Chemistry, Stanford University

[6]Innovative Medicines Accelerator, Stanford University

[7]Department Microbiology & Immunology, Stanford School of Medicine

[*]Correspondence to: dassama@stanford.edu

bioRxiv preprint doi: https://doi.org/10.64898/2026.07.06.735745; this version posted July 7, 2026. The copyright holder for this preprint (which was not certified by peer review) is the author/funder, who has granted bioRxiv a license to display the preprint in perpetuity. It is made available under a CC-BY 4.0 International license.

Supporting figures

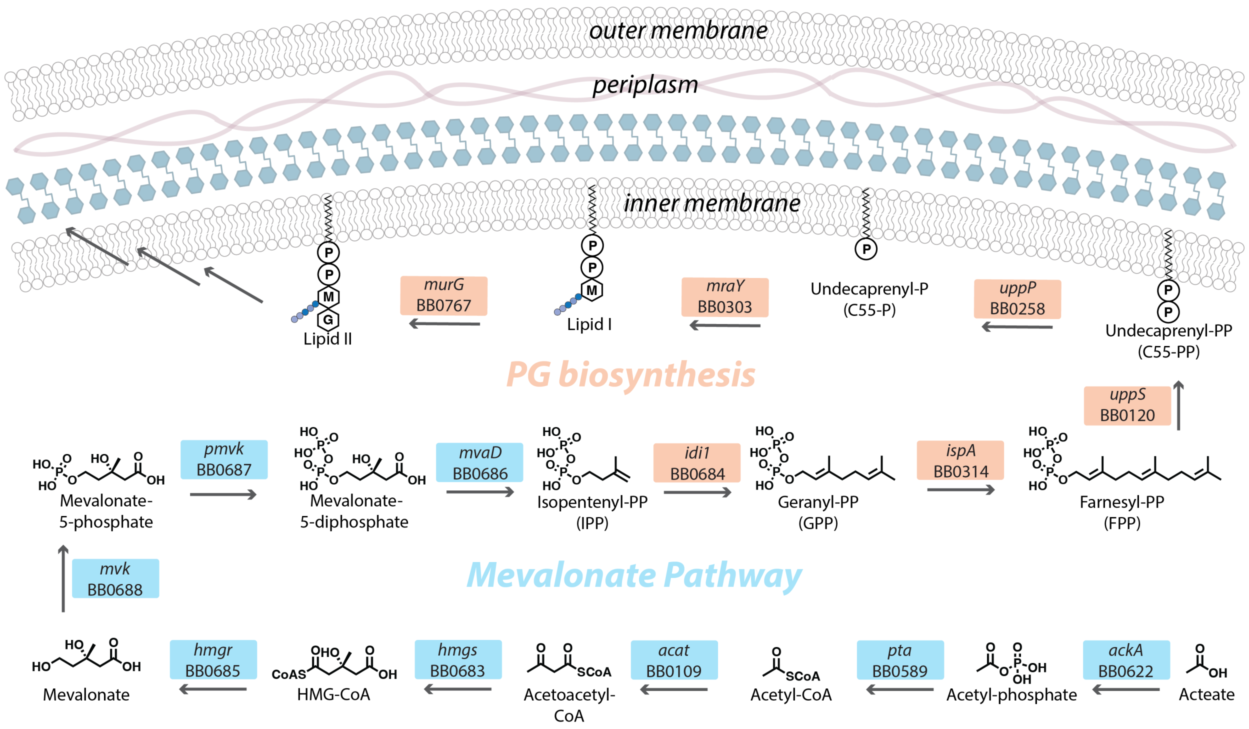

Fig. S1. *B. burgdorferi* B31 mevalonate and peptidoglycan (PG) biosynthesis pathways. Enzymes in the mevalonate and PG biosynthesis pathways from *B. burgdorferi* B31 identified by their locus tags and gene names. Also shown are the chemical structures of key molecules that they act on.

bioRxiv preprint doi: https://doi.org/10.64898/2026.07.06.735745; this version posted July 7, 2026. The copyright holder for this preprint (which was not certified by peer review) is the author/funder, who has granted bioRxiv a license to display the preprint in perpetuity. It is made available under a CC-BY 4.0 International license.

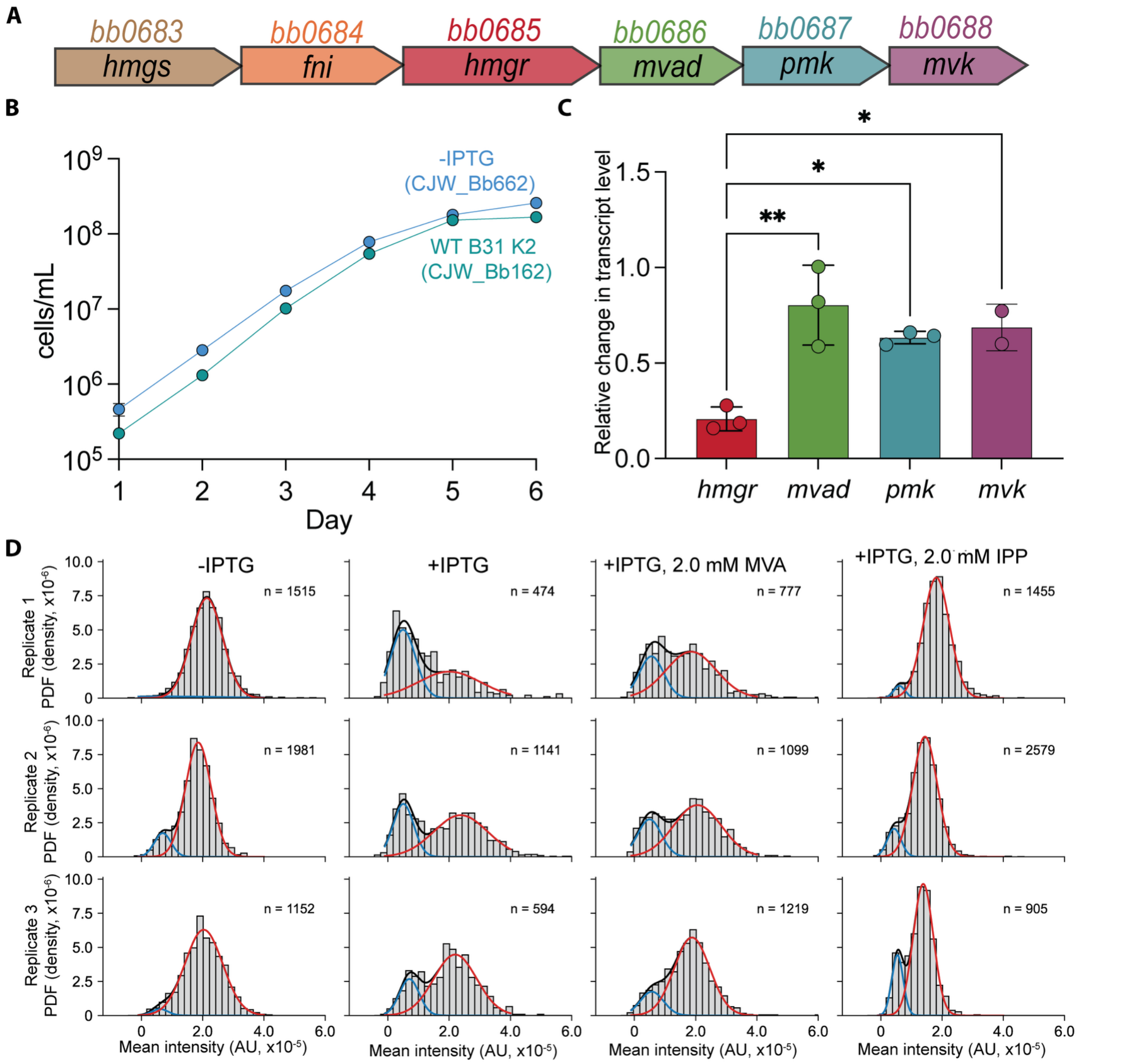

Fig. S2. Effects of CRISPR-interference of *hmgr* and HADA labeling statistics. A) Genome neighborhood diagram of the MVA pathway in *B. burgdorferi* strain B31. **B**) Growth curves of CJW_Bb662 in comparison to wild-type strain in the absence of IPTG. **C**) Plot showing the *fla*B-normalized transcript levels of the indicated gene under IPTG-inducing conditions relative to the noninducing (-IPTG) condition. CJW_Bb662 cultures grown in BSK-II to 1 x 10^7 cells/mL were split and either left untreated (-IPTG) or induced with 100 µM IPTG for 4 hours to trigger CRISPRi of *hmgr*. RNA was subsequently extracted from the samples and analyzed by qRT-PCR. Each dot represents a biological replicate. A one-way ANOVA with post-hoc Tukey test showed that the expression of *bb0686*, *bb0687*, *bb0688* is statistically different from that of

bioRxiv preprint doi: https://doi.org/10.64898/2026.07.06.735745; this version posted July 7, 2026. The copyright holder for this preprint (which was not certified by peer review) is the author/funder, who has granted bioRxiv a license to display the preprint in perpetuity. It is made available under a CC-BY 4.0 International license.

Full: Fig. S2. Effects of CRISPR-interference of *hmgr* and HADA labeling statistics. A) Genome neighborhood diagram of the MVA pathway in *B. burgdorferi* strain B31. **B**) Growth curves of CJW_Bb662 in comparison to wild-type strain in the absence of IPTG. **C**) Plot showing the *fla*B-normalized transcript levels of the indicated gene under IPTG-inducing conditions relative to the noninducing (-IPTG) condition. CJW_Bb662 cultures grown in BSK-II to 1 x 10^7 cells/mL were split and either left untreated (-IPTG) or induced with 100 µM IPTG for 4 hours to trigger CRISPRi of *hmgr*. RNA was subsequently extracted from the samples and analyzed by qRT-PCR. Each dot represents a biological replicate. A one-way ANOVA with post-hoc Tukey test showed that the expression of *bb0686*, *bb0687*, *bb0688* is statistically different from that of *bb0685* (*hmgr*) ($p < 0.05$) but not statistically different from each other ($p > 0.78$). **D**) Distributions of single-cell HADA intensities for individual biological replicates (related to Fig. 1G-J) across the indicated conditions. The cell number (n) for each Gaussian-fitted population is provided. The black lines represent the fit for the two populations whereas the red and blue lines show the contribution of each population.

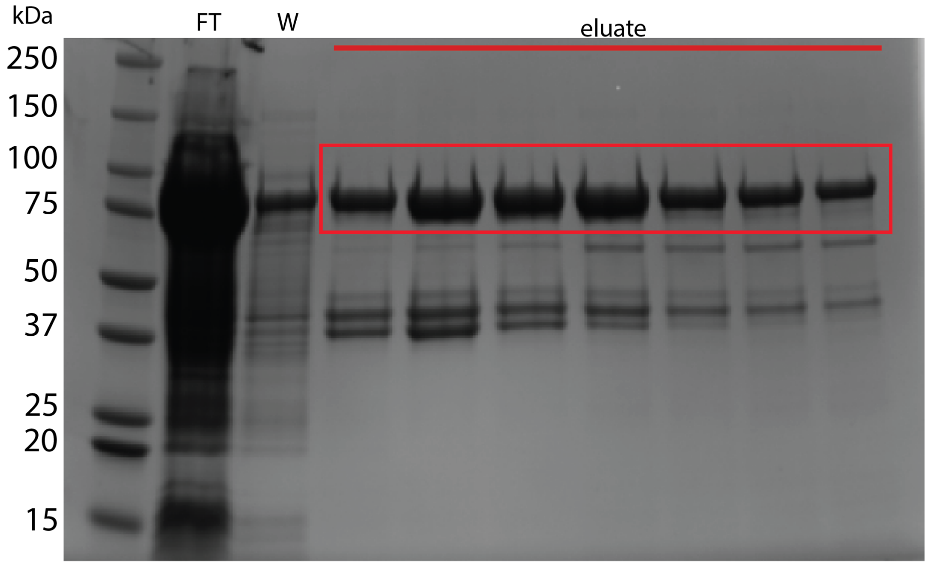

Fig. S3. Purification of *B. burgdorferi* HMGR. SDS-PAGE gel of MBP-HMGR following dextrin affinity chromatography. Molecular weight of the MBP fusion is ~91 kDa (red rectangle). FT: flow through; W: column wash.

bioRxiv preprint doi: https://doi.org/10.64898/2026.07.06.735745; this version posted July 7, 2026. The copyright holder for this preprint (which was not certified by peer review) is the author/funder, who has granted bioRxiv a license to display the preprint in perpetuity. It is made available under a CC-BY 4.0 International license.

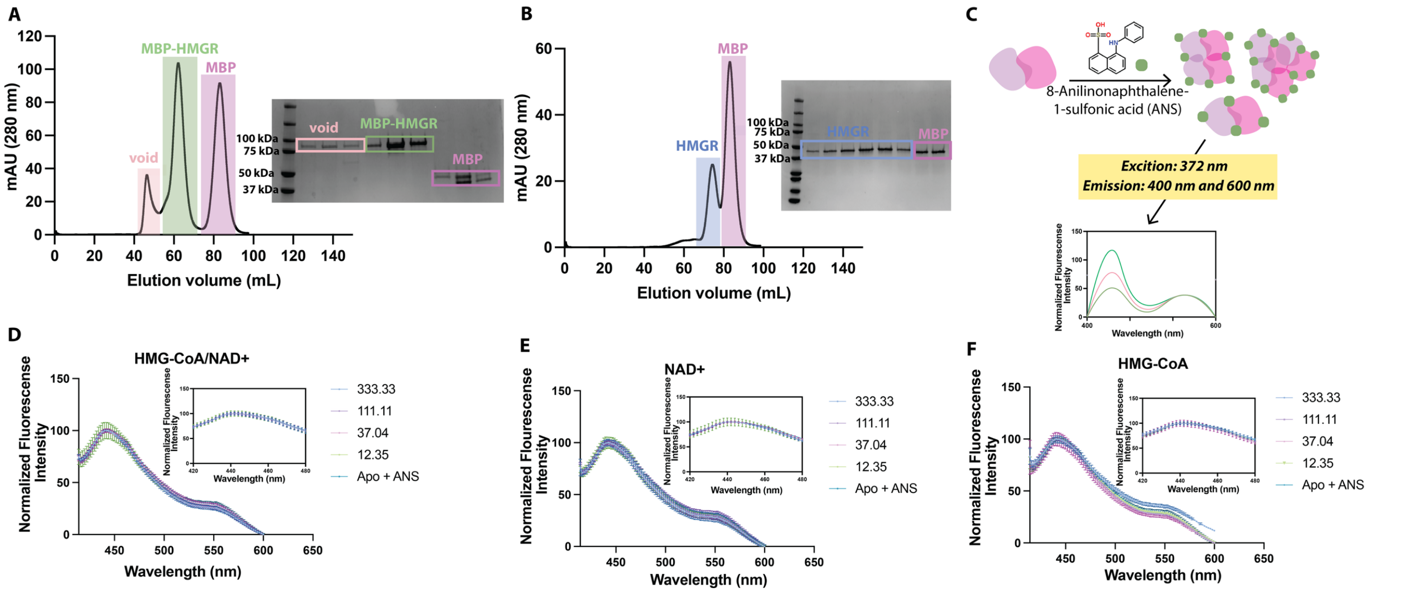

Fig. S4. Biochemical analysis of *B. burgdorferi* HMGR. **A, B)** Size-exclusion chromatography of *B. burgdorferi* MBP-HMGR fusion post amylose purification (**A**) and following Factor Xa cleavage of MBP (**B**). **C**) Schematic of ANS (1-anilino-8-naphthalene sulfonate) fluorescence assay to detect oligomerization. **D-F**) Fluorescence *vs* wavelength spectra with ANS probe and HMGR from *B. burgdorferi* with the addition of HMG-CoA, HMG-CoA/NAD$^+$, and NAD$^+$, respectively.

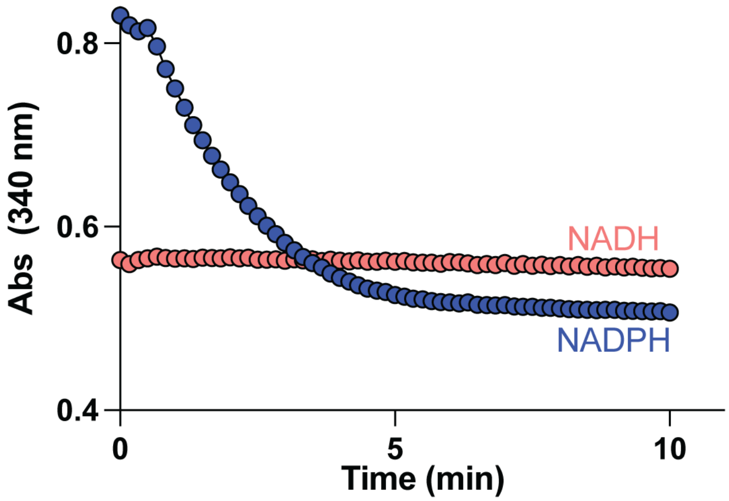

Fig. S5. Absorbance plot of *H. sapiens* HMGR with both cofactors. Absorbance plot over time of the *Homo sapiens* HMGR reaction monitoring the oxidation of NADH (red) or NADPH (blue). Assay conditions were 10 nM enzyme, 125 mM HMG-CoA, 250 NADPH or NADH.

bioRxiv preprint doi: https://doi.org/10.64898/2026.07.06.735745; this version posted July 7, 2026. The copyright holder for this preprint (which was not certified by peer review) is the author/funder, who has granted bioRxiv a license to display the preprint in perpetuity. It is made available under a CC-BY 4.0 International license.

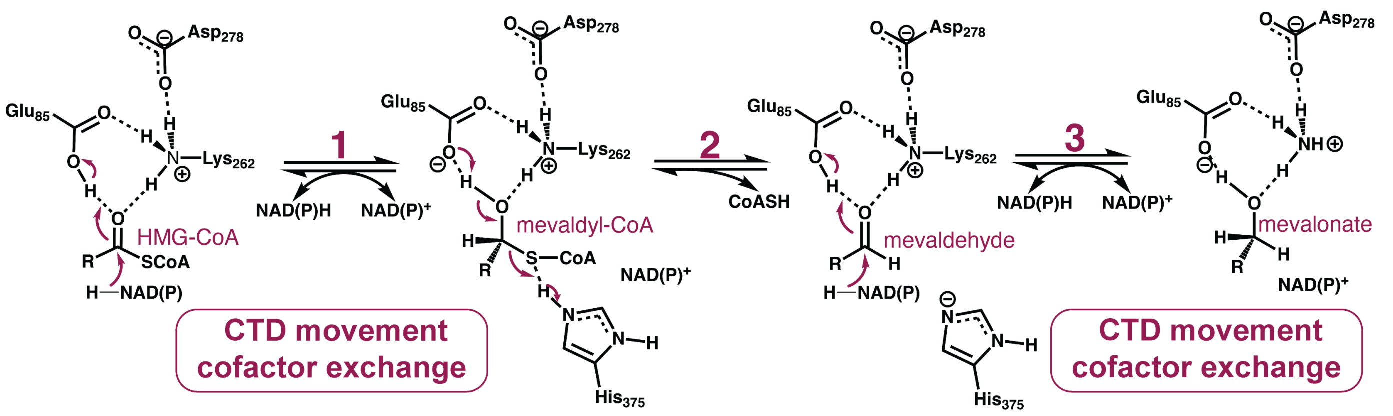

Fig. S6. Bacterial HMGR reaction mechanism. Abbreviated proposed reaction mechanism of HMGR from *B. burgdorferi*.

bioRxiv preprint doi: https://doi.org/10.64898/2026.07.06.735745; this version posted July 7, 2026. The copyright holder for this preprint (which was not certified by peer review) is the author/funder, who has granted bioRxiv a license to display the preprint in perpetuity. It is made available under a CC-BY 4.0 International license.

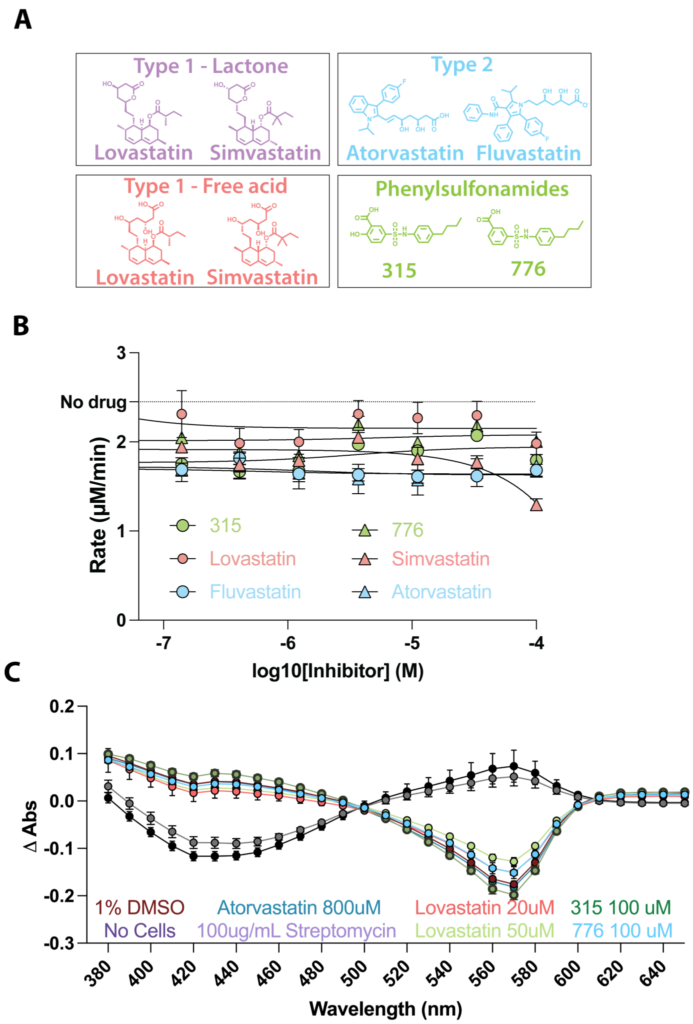

Fig. S7. Assessment of inhibitors targeting *B. burgdorferi* HMGR. A) Chemical structures of Type 1 and 2 statins and phenyl sulfonamides that inhibit the *E. faecalis* HMGR. **B**) Dose response for statins and phenyl sulfonamides 315 and 776 against purified *B. burgdorferi* HMGR. Assays were performed in triplicate using an absorbance-based assay and fit to log(inhibitor) versus response. **C**) Absorbance spectra of *B. burgdorferi* B31 K2 cultures (~1×10^7 cells/mL) grown in the presence of DMSO (diluent), streptomycin (known growth inhibitor), lovastatin at indicated concentrations (red and green), or atorvastatin (blue) using the pH indicator phenol red. Absorbance was measured every 10 nm from 350 to 650 nm. Absorbance values from day 0 were subtracted from day 8 to get Δ Abs which was plotted against the wavelength. Shown are the means and standard deviations for three technical replicates.

bioRxiv preprint doi: https://doi.org/10.64898/2026.07.06.735745; this version posted July 7, 2026. The copyright holder for this preprint (which was not certified by peer review) is the author/funder, who has granted bioRxiv a license to display the preprint in perpetuity. It is made available under a CC-BY 4.0 International license.

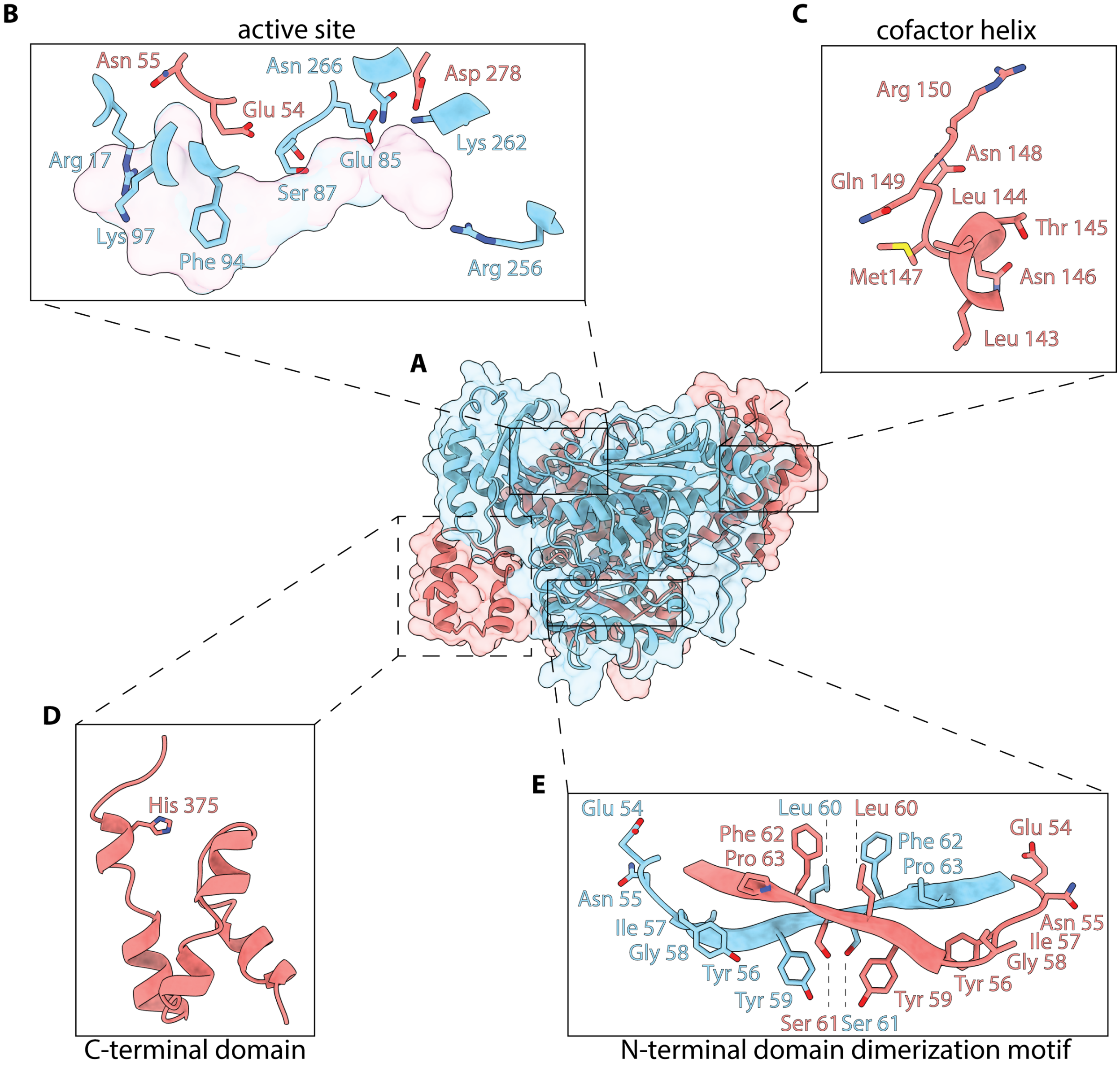

Fig. S8. **Crystallographic characterization of *B. burgdorferi* HMGR**: **A**) The homodimeric structure is depicted in both cartoon and a transparent surface format. One monomer is colored in blue while the other is shown in salmon. Several key functional domains are highlighted. **B**) Active site residues involved in binding substrate. **C**) Residues on the cofactor helix that engage with NAD(P)H in other bacterial HMGRs. **D**) The C-terminal domain with the catalytic His 375 shown in stick format. **E**) Residues that comprise the dimerization motif.

bioRxiv preprint doi: https://doi.org/10.64898/2026.07.06.735745; this version posted July 7, 2026. The copyright holder for this preprint (which was not certified by peer review) is the author/funder, who has granted bioRxiv a license to display the preprint in perpetuity. It is made available under a CC-BY 4.0 International license.

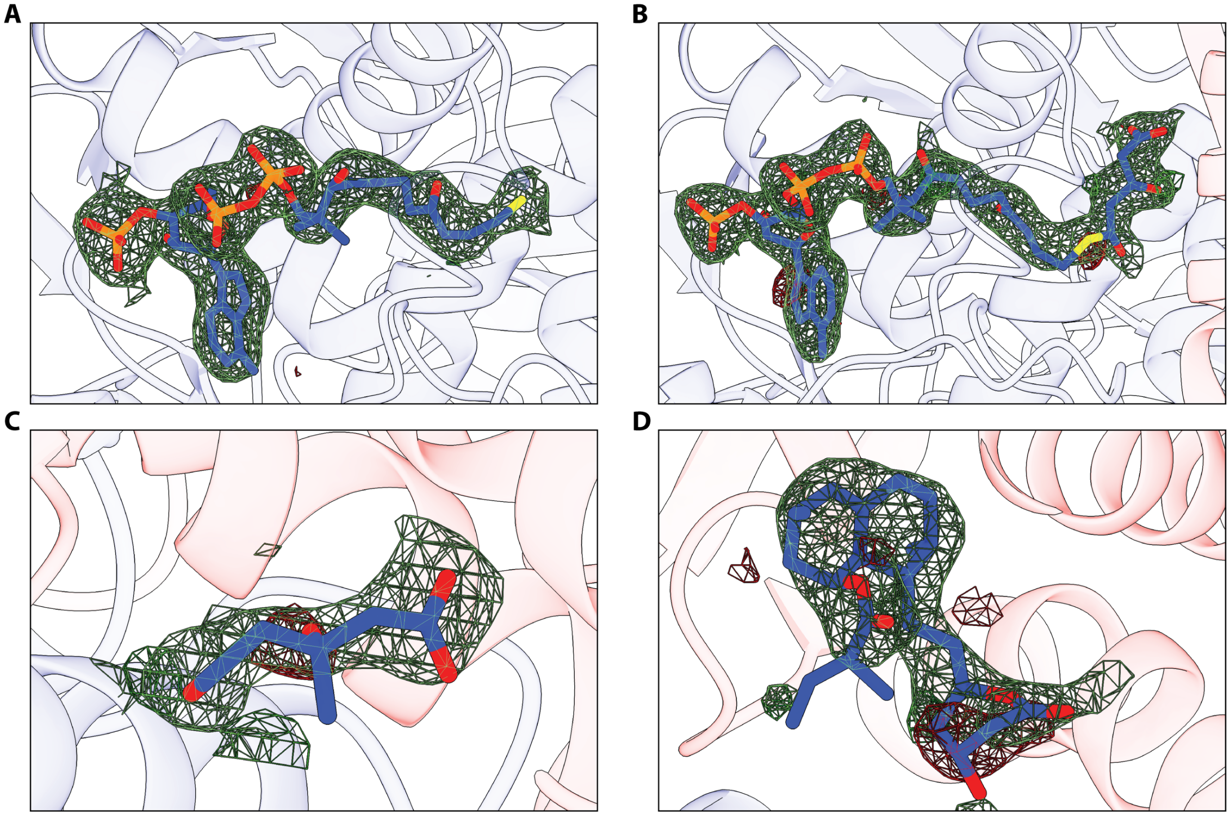

Fig. S9. Omit densities for *B. burgdorferi* HMGR ligands. mFo-DFc omit maps contoured at 3σ for **A**) CoA, **B**) HMG-CoA, **C**) mevalonate, and **D**) lovastatin bound to *B. burgdorferi* HMGR.

bioRxiv preprint doi: https://doi.org/10.64898/2026.07.06.735745; this version posted July 7, 2026. The copyright holder for this preprint (which was not certified by peer review) is the author/funder, who has granted bioRxiv a license to display the preprint in perpetuity. It is made available under a CC-BY 4.0 International license.

Supporting tables

Table S1. Fitting results for single cell analysis for the HADA labeling experiments.

Condition	P	μ_1	σ_1	μ_2	σ_2	replicate
- IPTG	0.06243371	69996.7541	196724.19	213737.496	51060.4125	1
- IPTG	0.11887534	69355.6573	27294.0446	185396.64	41926.3295	2
- IPTG	0.03133867	60144.1255	26171.7114	203320.872	61424.9433	3
+ IPTG	0.55158439	50085.1806	42309.1471	200194.443	90172.8925	1
+ IPTG	0.3269299	50529.271	33576.8089	237700.305	87884.5927	2
+ IPTG	0.21648618	69999.9875	32292.5845	219877.22	69952.5859	3
+ MVA	0.30393881	55442.7696	39546.1978	184999.97	81520.4471	1
+ MVA	0.26536664	49149.0514	38536.5146	205536.652	77275.86	2
+ MVA	0.16699063	56635.1894	38153.2122	187875.943	58114.2567	3
+ IPP	0.04149936	60380.0835	18321.4052	181148.631	43303.4658	1
+ IPP	0.11707549	43351.911	22654.2071	144038.835	39951.4086	2
+ IPP	0.20579118	54306.8701	18533.2712	138371.842	32820.1368	3

Table S2: Steady-state kinetics and inhibition of bacterial and human HMGRs

Enzyme	k_{cat} (min^{-1})	K_M (µM)	IC$_{50}$ values (µM)				
			Lovastatin	Simvastatin	Fluvastatin	Atorvastatin	Cambridge 315
H. sapiens[29]	840	2.5	0.023	0.011	0.028	0.008	>200
E. faecalis[28]	66	20	>200	>200	N/A	N/A	7.1
B. burgdorferi	2.1	7.7	>200	>200	>200	>200	>200

Table S3: Bacterial strains used in this study

B. burgdorferi strains

Strain	Relevant genotype/description	Antibiotic resistance	Source
B31 MI	Infectious, mouse isolate	-	(PMID 9403685)
B31 A3	B31 MI *cp9*$^{-}$	-	(PMID 11895980)
B31K2	B31-A3-68-*bbe02*::P$_{flgB}$-*aphI*, *lp56*$^{-}$, *cp9*$^{-}$	Kan	(PMID 21193609)
CJW_Bb662	B31 K2/pBbCas9S(RBSmut)-P$_{syn}$-sgRNA^{BB0685} *lp56*$^{-}$, *cp9*$^{-}$	Kan, Strep	This work

E. coli strains

bioRxiv preprint doi: https://doi.org/10.64898/2026.07.06.735745; this version posted July 7, 2026. The copyright holder for this preprint (which was not certified by peer review) is the author/funder, who has granted bioRxiv a license to display the preprint in perpetuity. It is made available under a CC-BY 4.0 International license.

NEB C2992H	*F′ proA+B+ lacIq Δ(lacZ)M15 zzf::Tn10 (TetR) / fhuA2Δ(argF-lacZ)U169 phoA glnV44 Φ80Δ(lacZ)M15 gyrA96 recA1 relA1 endA1 thi-1 hsdR17*	-	
CJW7166	CJW_7551/pBbdCas9S(RBSmut)_P_{syn}-sgRNA500	Strep	(PMID 33257311)
CJW7658	CJW_7551/pBbCas9S(RBSmut)-P_{syn}-$sgRNA^{BB0685}$	Strep	This work

**Table S4. Bacterial plasmids used in this study**			
**Plasmid**	**Description**	**Antibiotic resistance**	**Source**
pBbdCas9S(RBSmut)_P_{syn}-sgRNA500	Parent for the "all-in-one" CRISPRi shuttle vector. It has a mutation in the RBS to help reduce leakiness of the dCas9 expression.	Strep	(PMID 33257311)
pBbCas9S(RBSmut)-P_{syn}-$sgRNA^{BB0685}$	"All-in-one" shuttle vector that includes an sgRNA targeted against *bb0685*.	Strep	This work

Protein sequences

Borreliella burgdorferi (strain ATCC 35210 / DSM 4680 / CIP 102532 / B31)
Uniprot: O51628

MNLESLSSFMELSKNFRHKSVLEKRQEIKSFLELSYKDFFYNNANEDFLFNMIENYIGYL
SFPIGIVKNLKINGKYYSLPIATEESSVVAALNFAAKILENADLRYSLGEVLGISQIYIKSE
KDLSKIFVDLGDKIKTWIEPLLTNMNQRGGGFRRLSTRHIKELGIQKLNIYVDTCDAMGA
NLLNSIAERVAEFIFLEFGYECVLKVLSNDISEFTAKARFVLDFKHLLPGKEDSWNLAKKI
ELISSIGFYEEERAVTNNKGIMNGITGVCLATFNDTRALEASVHKFASKSGKYFPLSKFYT
TDNALVGEIEIPLQVG
TKGGVISFNEASILSFKIMNVNSKSEFIGILSCVGLASNFAALRALAFNGIQKGHMRLHVN
KILHLLKTKYNISDFEKDKLLLEMERMNIYSFDFAFKILKKIRLENENKV

Lactobacillus jensenii
Uniprot: A0A5N1IG17

MKFYQLPISERRKMLLQNGIKLNHVDDDLLSELDLLSENVIGKLTLPLSVLQTAIVNGQS
FQVPMATEESSVVAAANHGLNIFNQNGGVSAKSERTGIWGQLVFEVAEFSLAEFEAKKP
DYLKLVNEEFASLVKHGGGVRQIIAEVKTDLLFLRVLVDPAESMGANRTNTILEFLGQKI
SQDFTIEKLYAILSNYPSQYTCAKVSLAFASLTKTKDEKIGEKIAQKIVLLSKIGQEDPYR
AVTNNKGIMNGVDAILLATGNDFRAVEAACHQAASLSGSYQSLSNWRIEDNKLVGEIK
LPLAIGVVGGSIKSRSDVQVAYRILRQVTASELAEIIAAVGLANNLAALLAISTVGIQKGH
MSLQIRNVLKNLTATDEEKNSVKELMQKQKRYSETDAKKFLQEIREENN

Flavobacterium psychrophilum
Uniprot: A0A076P6Q4

bioRxiv preprint doi: https://doi.org/10.64898/2026.07.06.735745; this version posted July 7, 2026. The copyright holder for this preprint (which was not certified by peer review) is the author/funder, who has granted bioRxiv a license to display the preprint in perpetuity. It is made available under a CC-BY 4.0 International license.

MPKLTTGFSKLSKEEKINWIASTHFSNAEEATQTIKKYWNSDLELQKLHDEFIENTITNFY
LPLGVAPNFLINGKNHTIPFAIEESSVVAAASKSAKYWGTRGGFKTTVLNSEKIGQVHFIF
KGDSRNLIVFFNQIKSKLFAHTESITTNMQKRGGGILDIELRDKTSDLENYYQLHATFETK
DSMGANFINSCLEQFAKTLKEEALQSEILSETEKNIEVVMSILSNYVPNCIVRAEVSCPVS
DLSEKNIENPQEFAQKFIRAVKIAEIEPFRAVTHNKGIMNGIDAVVLATGNDFRAVEAGI
HAYAARNGQYSSLSHAKIENDIFTFWLEIPLALGTVGGLTSLHPLVKMSLEMLEKPSAQE
LMQIVAVAGLAQNFAALRSLTTTGIQQGHMKMHLNNIINQFEATQNERVLIKNHFTENT
VSHSAVVAFIESLRK

Legionella pneumophila subsp. pneumophila (strain Philadelphia 1 / ATCC 33152 / DSM 7513)
Uniprot: Q5ZTV6

MSIAANASELFRGFSKLSREERFQRLCALGALTHEDITFLKQGGIKDLNLADKLIENVIGY
FQLPLGVATNFNIDGRDYVIPLAVEETSIIAALSKSAKWIRQHGEINTWVHGECILGQIQL
AKVKDFQRFSDLFNKNRQYFIEIANKDVAANMVKRGGGVTDLQVRHLKREDGLDMAV
IHLTMNSCDAMGANIINQVLEYLKQPIEQITGEEVTMCILSNLNDQKLTTAQVIIRNIDPIL
GQKLQEASLFAEIDPYRAATHNKGVMNGIDPVLIATGNDWRAVEAGIHAYAARSGQYK
AITRWRYQNEILTGELTAPIIVGTVGGVTSLHPTAKMCLRMMDITSANQLSQVIAAVGLV
QNLGALKALCTDGIIQGHMKLHIDNLLLVAGANENEMPVLKEKLQEWLNLNKRVSLNN
AYDLLAEIRQAPVAV

bioRxiv preprint doi: https://doi.org/10.64898/2026.07.06.735745; this version posted July 7, 2026. The copyright holder for this preprint (which was not certified by peer review) is the author/funder, who has granted bioRxiv a license to display the preprint in perpetuity. It is made available under a CC-BY 4.0 International license.

[#]Also affiliated with: CADD Consulting GmbH
